# Editorial: Adrenal related hypertension: from bench to bedside, volume II

**DOI:** 10.3389/fendo.2025.1647120

**Published:** 2025-07-03

**Authors:** Amnani Aminuddin, Elena Aisha Azizan

**Affiliations:** ^1^ Department of Medicine, Faculty of Medicine, Universiti Kebangsaan Malaysia, Kuala Lumpur, Malaysia; ^2^ Research Center, Hospital Tunku Ampuan Besar Tuanku Aishah Rohani, Universiti Kebangsaan Malaysia Specialist Children’s Hospital, Kuala Lumpur, Malaysia

**Keywords:** endocrine hypertension, adrenal, aldosterone, primary aldosterone, hypertension, secondary hypertension

Hypertension secondary to adrenal disorders remains a major risk factor for cardiovascular diseases and death (1, 2). Primary aldosteronism (PA), the most common form of adrenal related hypertension, is frequently asymptomatic and underdiagnosed, highlighting the need for improved screening and diagnostic strategies (3). Furthermore, understanding the causes is key to improving its management (4, 5). This Research Topic compiles 4 review articles and 8 original research articles that explore new diagnostic and prognostic approaches, as well as new insights into the pathogenesis of adrenal related hypertension.

While current PA guidelines recommend measuring the plasma ARR, the prevalence of positive screening varies due to unstandardized protocols (6). The discovery of aldosterone driver mutations in both normotensive (in aldosterone-producing micronodules of normotensive adrenal glands that increase with age) and hypertensive individuals, suggest that PA exists along a continuum of disease progression rather than being limited to distinct subtypes (7). In light of these, Kitamoto et al. reviews the need to refine current strategies for screening PA to improve detection and address underdiagnosis. They correlate low renin levels with increased cardiovascular risk from dysregulated aldosterone production. Accordingly, they propose that optimal screening should be conducted in all hypertensive patients and begin with plasma renin activity (PRA) evaluation, using a low PRA (<1.0 ng/mL/h) and high plasma aldosterone concentration (PAC; >12 ng/dL).

Our review (Aminuddin et al.) summarizes current knowledge on adrenal cortex homeostasis regulation, offering insights into PA pathogenesis and the rationale for using aldosterone synthase (CYP11B2) inhibitors as treatments for patients who are not candidates for surgery or have experienced surgical failure. The review identifies key factors disrupting adrenocorticoids homeostasis, including aldosterone driver mutations, contributing to the development of CYP11B2-positive adrenal cortical neoplasms. Given the key role of CYP11B2 in PA, the review compiles pharmacological strategies targeting this enzyme and explores how CYP11B2 inhibition impacts adrenal cell fate to ensure the safety and efficacy of the treatments.

Xiang et al.’s comprehensive review, discusses key factors that may predict the prognosis following adrenalectomy. This is especially important for closer monitoring in patients with a poorer prognosis. Preoperative factors such as lower body mass index, female sex, younger age, shorter duration of hypertension, larger nodule on imaging, and low periadrenal adipose tissue volume are associated with clinical success. Variant adrenal venous anatomy complicates adrenalectomy. The classical type of unilateral PA (UPA) had a better prognosis compared to the non-classical type. Lastly, genetic features may have variable effects on prognosis.

In the systematic review McCarthy et al., the authors selected three single-center studies from Japan, Singapore, and China to determine the prevalence of PA among patients with stroke or transient ischemic attack (TIA). From the meta-analysis, the pooled PA prevalence in adults with stroke or TIA is not uncommon with 5.8%. Though, the statistical heterogeneity was high, with an I^2^ statistic of 87.6%. This heterogeneity could be due to differences in patient age, the timing of PA testing after acute cerebral event, aldosterone-to-renin ratio (ARR) thresholds, and confirmatory testing methods, as well as the confounding effects of antihypertensive drugs.

Interestingly, Jiang et al. reported that UPA with cortisol co-secretion is not uncommon in the Chinese population. Notably, the combination of excess aldosterone and cortisol increases the risk of cardiovascular diseases compared to UPA without cortisol secretion, underlining the need for corticol co-secretion screening and appropriate management of UPA with cortisol co-secretion to optimize patient outcomes. They found that UPA patients with cortisol co-secretion had distinct clinical characteristics and had a lower chance of achieving complete clinical success. They were older, had a longer history of hypertension, larger adrenal tumors, and were more responsive to ACTH.

Meanwhile, Sun et al. compared the diagnostic efficacy of the saline infusion test and captopril challenge test (CCT) for PA. Their findings suggest that CCT had higher diagnostic value based on post-CCT PAC suppression. Importantly, the optimal cutoff for post-CCT PAC suppression differed for patients under and over 50 years old in the Chinese population. This supports the need for personalized diagnostic approaches in PA.

Whereas the original research by Yin et al. reported that the non-invasive ^68^;Ga-Pentixafor Positron Emission Tomography/Computed Tomography (PET/CT) procedure was an efficient method for diagnosing PA compared to adrenal vein sampling (AVS) with sensitivity 89% vs. 79%. Moreover, the PET/CT identified 94% of patients who achieved complete biochemical and clinical success after adrenalectomy, compared to 78% identified by AVS, suggesting enhanced predictive accuracy of PET/CT. Of note, cases with unclear subtyping diagnoses based on AVS results were included in this study. Yin et al.’s findings is supported by another study in a Chinese cohort, that found ^68^Ga-pentixafor PET/CT to identify the dominant side of aldosterone secretion in PA with an accuracy rate similar to that achieved by AVS (85.7% vs. 71.4%) (8).

In a retrospective study, ter Haar et al. suggested a clinical decision model to subtype PA, particularly when right adrenal vein cannulationin AVS is unsuccessful. They proposed a decision index with a specificity exceeding 90% by using the ratio of aldosterone to cortisol from the left adrenal vein (LAV) and the inferior vena cava (IVC). According to their model: (1) an LAV/IVC index <1.2 suggests unilateral right-sided PA and supports right adrenalectomy; (2) a ratio between 1.2 and 2.4 indicates bilateral disease, thus mineralocorticoid receptor antagonist (MRA) therapy is advised; (3) an index ≥4.4 suggests unilateral left-sided PA, indicating left adrenalectomy; and (4) a resampling is advised only when the index is between 2.4 and 4.4.

In context of pathophysiological of PA, Nanba et al. investigated the potential association of double *CTNNB1* and *GNA11/Q* mutations in aldosterone-producing adenomas with pregnancy, menopause or puberty through a case study of a Japanese female patient with UPA, who also had high mRNA expression of luteinizing hormone/choriogonadotropin receptor (*LHCGR*) and gonadotropin-releasing hormone receptor (*GNRHR*). Despite experiencing menopause-like symptoms, the patient had regular menstrual cycles and no history of pregnancy-induced hypertension. They concluded that the disease can occur without a clear association with pregnancy or menopause.

Recent studies support the need to revisit the concept of mild PA (6). Herein, between 2017 – 2022, Makhnov et al. performed a large-scale screening for PA in an unselected cohort of primary care patients with hypertension in Sweden, aged 18–65 years according to the Endocrine Society guidelines (9). Among 1181 recruited patients, the PA prevalence among hypertensive patients was 4.5%, consistent with range reported in the Endocrine Society Guidelines (~ 5 – 13%). Importantly, they observed that the ARR≥50 pmol/mIU as an optimal diagnostic cut-off and recommended routine PA screening in hypertensive patients to improve patient management and reduce risk of morbidity and mortality.

The significant role of non-defining adrenal steroids in the metabolic alterations and comorbidities observed in endocrine hypertension, including pheochromocytoma/paraganglioma (PPGL), Cushing’s syndrome, and PA was investigated by Knuchel et al. study. The retrospective analysis of 263 patients revealed that metabolomic profiles are not only influenced by disease-defining hormones, but also by other adrenal steroids. In PPGL, metabolomic changes were driven by catecholamine excess. In Cushing’s syndrome, cortisol along multiple non-defining adrenal hormones contributed to metabolomic variation. In PA, non-aldosterone steroids like cortisol, cortisone, and dehydroepiandrosterone showed stronger associations compared to aldosterone.

Finally, Raber et al.’s study involving 303 patients with PPGL assessed long-term and survival outcomes. They found that overall survival and disease-specific survival (DSS) were generally favourable, especially in non-metastatic cases. Only 5% of all deaths were directly attributed to PPGL, with the remaining deaths caused by cardiovascular disease and other malignancies. Patients with metastases at diagnosis had the poorest outcomes, while those with non-metastatic recurrences had much longer survival. Major adverse cardiovascular events occurred before diagnosis in 15% of patients and were strongly associated with shorter survival. Predictors of shorter DSS include older age, male sex, history of major adverse cardiovascular events, and primary metastatic disease.

In summary, this Research Topic highlights the evolving adrenal hypertension research, focusing on precise diagnostics, molecular analysis, and physiologically-informed screening to improve clinical care. The need for more personalized, evidence-based approaches is crucial. Whereas multi-center studies are needed to validate emerging screening strategies and therapies.
